# TlyA is a 23S and 16S 2′-O-methylcytidine methyltransferase important for ribosome assembly in *Bacillus subtilis*

**DOI:** 10.1093/nar/gkaf1531

**Published:** 2026-01-15

**Authors:** Jennie L Hibma, Lia M Munson, Joshua D Jones, Taylor M Nye, Kristin S Koutmou, Lyle A Simmons

**Affiliations:** Department of Molecular, Cellular, and Developmental Biology, University of Michigan, Ann Arbor, MI 48109, United States; Department of Molecular, Cellular, and Developmental Biology, University of Michigan, Ann Arbor, MI 48109, United States; Department of Chemistry, University of Michigan, Ann Arbor, MI 48109, United States; Department of Molecular, Cellular, and Developmental Biology, University of Michigan, Ann Arbor, MI 48109, United States; Department of Chemistry, University of Michigan, Ann Arbor, MI 48109, United States; Department of Molecular, Cellular, and Developmental Biology, University of Michigan, Ann Arbor, MI 48109, United States

## Abstract

Ribosomal RNA (rRNA) methylation is conserved across biology, yet the effect of rRNA methylation on ribosome function is poorly understood. In this work, we identify a biological function for the rRNA 2′-O-methylcytidine methyltransferase TlyA, conserved between *Bacillus subtilis* and *Mycobacterium tuberculosis (Mtb)*. The *tlyA* deletion in *B. subtilis* confers a cold sensitive phenotype and resistance to aminoglycoside and cyclic polypeptide antibiotics. We show that ∆*tlyA* cells have ribosome assembly defects characterized by accumulation of the 50S subunit. Using a genetic approach, we tested the importance of potential catalytic residues and S-adenosyl-l-methionine (SAM) cofactor binding sites identified based on sequence alignments with other rRNA methyltransferases. We show that *B. subtilis* TlyA uses the common rRNA methyltransferase catalytic triad KDK and SAM binding motif GxSxG. This differs from TlyA from *Mtb*, which requires an additional tetrapeptide linker. Together our work demonstrates that *B. subtilis tlyA* is critical for ribosome assembly and we identify key residues for TlyA function *in vivo*. Since *Escherichia coli* lacks TlyA or a functional equivalent, our work highlights key differences in ribosome maturation between *B. subtilis, Mtb*, and more divergent Gram-negative bacteria providing new insight into rRNA maturation and antibiotic resistance mechanisms.

## Graphical Abstract

**Graphical Abstract:**
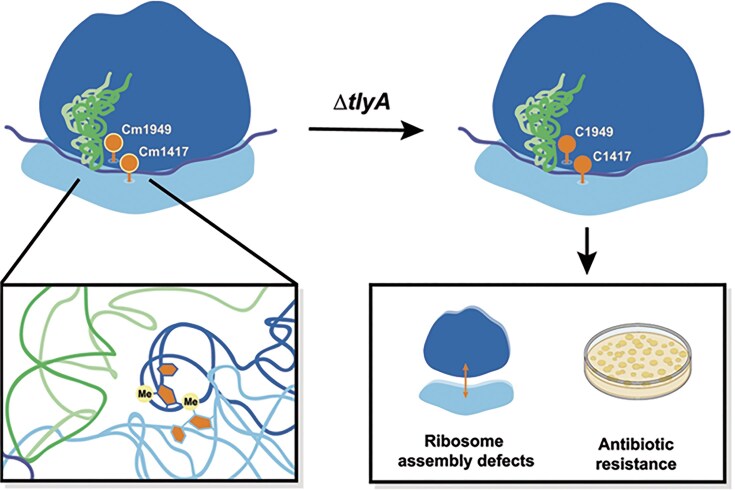

## Introduction

Ribosomes are one of the most abundant, complex, and efficient ribonucleoprotein structures found in the cell [1]. They function by translating messenger RNA (mRNA), transcribed from coding sequences in genomic DNA (gDNA), into polypeptides for use in virtually all cellular functions [2]. Mature ribosomes decode mRNA at a rate of ~50 nucleotides/second (nt s^−1^) [3, 4]. To translate proteins with such high speed and accuracy, proper ribosome architecture must be efficiently achieved and maintained.

The first step toward attaining functional ribosome architecture in bacteria is accomplished by a series of endonucleases that cleave ribosomal RNA (rRNA) transcripts that are transcribed together from multiple *rrn* operons in the bacterial chromosome [5, 6]. The cleaving of intergenic regions yields mature 16S, 23S, and 5S rRNAs. Each rRNA then folds independently or with the help of ribosome biogenesis factors to prevent misfolding and ensure assembly with the appropriate ribosomal proteins (r-proteins) [7, 8]. In addition to folding and binding r-proteins, rRNA maturation also includes the addition of posttranscriptional modifications. rRNA modifying enzymes, predominantly methyltransferases (MTases) and pseudouridine synthases, catalyze the chemical modification of rRNA nucleobases or ribose sugars [9, 10]. rRNA MTases methylate either the 2′-hydroxyl group of the ribose sugar or the nitrogenous base. S-adenosyl-l-methionine (SAM) is used to transfer a methyl group to their target substrate recognized by sequence and secondary structure [9, 11]. Methylations are important for formation of functional ribosomal subunits and act to enhance rRNA folding by creating steric hindrance and hence are an important factor in the formation of functional ribosomal subunits [12]. The secondary structure of rRNA is important for the cooperative binding of r-proteins and formation of the peptidyl transferase catalytic center of the ribosome for efficient mRNA decoding [13]. Indeed, many modifications are clustered in the functionally important regions of the ribosome used for decoding, peptidyl transfer, transfer RNA (tRNA) binding, and the peptide exit tunnel [14, 15]. Through the cumulative actions of these proteins, the final mature bacterial ribosome (70S) is formed composed of the large subunit (50S) containing 23S and 5S rRNAs and 33 r-proteins and the small (30S) subunit containing the 16S rRNA and 21 r-proteins [16].

Much of our knowledge on bacterial rRNA methyltransferases and their modifications come from studies in the Gram-negative organism *Escherichia coli. Escherichia coli* ribosomes possess 24 rRNA methylations incorporated by 23 different methyltransferases [17]. Comparatively, little is known about rRNA modification in Gram-positive bacteria [14, 18–22]. The first full profile of rRNA modifications was only recently reported for a Gram-positive bacterium, *B. subtilis* [23]. However, while the location of modified sites has been reported, in contrast to *E. coli*, the identity of the enzymes responsible for most *B. subtilis* rRNA modifications remain to be identified. Indeed, only five *B. subtilis* rRNA methyltransferases have been identified so far including RsmG, RlmCD, RlmP, RlmQ, and TlyA [23–27]. Furthermore, the contributions of these *B. subtilis* rRNA modifications to ribosomal maturation and function remains elusive, as only two of these RlmP and RlmQ have been phenotypically characterized [26, 27].

In this work, we characterize the dual-substrate 16S and 23S 2′-O-methylcytidine (Cm) methyltransferase TlyA, previously named YqxC, in *B. subtilis*. This enzyme was renamed TlyA in *B. subtilis* (TlyA*_Bs_*) after TlyA in *Mycobacterium tuberculosis* (TlyA*_Mtb_*). TlyA*_Mtb_* functions equivalently as a 16S/23S 2′-O-methylcytidine methyltransferase and modifies orthologous positions on the *M. tuberculosis* rRNAs though it does not follow rRNA methyltransferase naming conventions because it was originally annotated as a hemolysin [22, 28, 29]. Out of the known *B. subtilis* enzymes, only RlmP and TlyA have been shown to be 2′-O-methyltransferases, and RlmP does not have any known deletion phenotypes [26]. *Escherichia coli* contains four 2′-O-methyltransferases including three that modify the 23S as Gm2251, Cm2498, and Um2552, conferred by RlmB, RlmM, and RlmE, respectively [30, 31, 32]. Only RlmE mutants demonstrate a delay in 50S ribosome assembly and compromised translation initiation and elongation [32, 33]; RlmB and RlmM show slight reductions in fitness, but the contribution of methylation to ribosome assembly is unclear [30, 31]. On the *E. coli* 16S rRNA, C1402 is dimethylated by RsmI and RsmH forming Cm and m^4^C modifications, respectively, to shape the P site of the ribosome and assist in mRNA decoding accuracy [34]. Therefore, Nm modifications have been shown to be important for ribosome assembly and translational fidelity in *E. coli*, but their contribution in *B. subtilis* remains unknown.

We describe the biological consequences of the deletion of *tlyA*, finding that ∆*tlyA* cells exhibit cold sensitivity and resistance to aminoglycoside, and cyclic peptide antibiotics. Cold sensitivity can be attributed to a ribosome assembly defect, as shown by increased levels of 50S large subunits in ∆*tlyA* cells. We also determine the identity and location of the modifications conferred by TlyA*_Bs_* as Cm modifications at C1949 of the 23S rRNA and C1417 of the 16S rRNA that was done concurrently with the work presented in [23]. Additionally, we identify the catalytic site and SAM cofactor binding domain of TlyA*_Bs_ in vivo*, providing insight into the mechanism used to deliver methyl groups to two cytosine residues in the 16S and 23S rRNA. Importantly, we note that deletion of TlyA*_Bs_* cannot be complemented with its ortholog from *M. tuberculosis* suggesting differing enzyme structure between the two species. Taken together, this work characterizes the first and only identified rRNA methyltransferase in *B. subtilis* that modifies both the 16S and 23S rRNAs. Our work also highlights a key evolutionary difference between Gram-positive and Gram-negative bacteria. Some Gram-negative bacteria have a Type I TlyA (TlyA^I^), but these enzymes only modify C1920 in the 23S rRNA. In contrast the Gram-positive *B. subtilis* and diderm *M. tuberculosis* contain the Type II (TlyA^II^) versions that modify both the 23S and 16S [35, 36]. Interestingly, other Gram-negative species, notably *E. coli*, completely lack both a Type I or Type II TlyA enzyme making studies of Gram-positive TlyA^II^ important for understanding ribosome assembly and antibiotic resistance mechanisms in environmentally and clinically relevant Gram-positive bacteria [35].

## Materials and methods

### Strains and media

Bacterial strains were all derived from *B. subtilis* lab strain PY79. Strains used, primers, and plasmids are listed in Supplementary Tables S1–S3. *Bacillus subtilis* strains were regularly grown in LB broth (10 g/l tryptone, 10 g/l NaCl, 5 g/l yeast extract). LB agar used to plate strains contained the same ratio but with 15 g of agar supplemented. Plates and cultures were grown regularly at 37°C instead of 30°C to mitigate the cold defect seen in ∆*tlyA*. When necessary, broth or plates were supplemented for 100 µg/ml spectinomycin or 0.5 µg/ml erythromycin for selection. Freezer stocks of bacterial strains were composed of 900 µl of culture grown to OD_600_ > 1.0 and 100 µl dimethyl sulfoxide and stored at −80°C.

### ∆tlyA strain construction and transformation

*Bacillus subtilis yqxC::loxP-erm-loxP trpC2* strain was obtained from the Bacillus Genetic Stock Center [37]. gDNA was isolated from this strain and and used to transform competent WT cells. To make competent, WT bacteria was inoculated into LB supplemented with 3 mM MgSO_4_ and grown on a rolling rack at 37°C for ~3 h or until reaching an OD_600_ of > 1.0. Cultures were back diluted 1:25 into 500 µl MD media [1× PC buffer (10× PC buffer: 107 g/l K_2_HPO_4_, 60 g/l KH_2_PO_4_, 10 g/l trisodium citrate, 2% glucose, 50 µg/ml tryptophan, 50 µg/ml phenylalanine, 11 µg/ml ferric ammonium citrate, 2.5 µg/ml potassium aspartate, 3 mM MgSO_4_)] and grown for 4 h on a rolling rack at 37°C. No more than 500 ng DNA was added to the competent culture and transformed for 1–1.5 h on a rolling rack at 37°C. Transformation was confirmed using antibiotic selection and polymerase chain reaction (PCR). Subsequent clean deletion made use of the Cre recombinase expressing plasmid pDR244 as described previously [38].

### ∆tlyA complementation

For genetic complementation, we cloned *tlyA* or mutant *tlyA* into the modified version of plasmid pDR110 (pPB194) containing the P_hyperspank_ promoter and spectinomycin selection cassette flanked by *amyE* regions. To do this, our gene of interest was amplified with primers containing overhanging complementary regions to our plasmid. For *tlyA_Mtb_*, a gene block was ordered from Twist Bioscience using the *Mtb* TlyA amino acid sequence with *B. subtilis* codon optimization and an N-terminal 3X-Myc tag. These were combined with amplified plasmid backbone and Gibson assembled according to manufacturer’s recommendations (NEB E2611). The Gibson reaction was then used to transform MC1061 *E. coli*, successful transformants selected by ampicillin, and mini-prepped according to manufacturer’s instructions (Qiagen 27104). Miniprepped plasmid sequences were confirmed with Sanger sequencing. Following, *∆tlyA B. subtilis* was made competent and transformed as described above resulting in homologous recombination at the *amyE* locus. This disruption of the *amyE* gene was confirmed by the strain’s inability to metabolize starch by growth on an LB agar plate containing 10 g/l starch and then stained with solid iodine. Strains with successful recombination as indicated by failure to metabolize starch underwent PCR of the *amyE* locus to ensure correct variant length of the disrupted locus.

### Genomic DNA purification

Desired strains were struck out onto the appropriate LB agar plates and incubated at 37°C overnight. A single colony was used to inoculate 5 ml LB media, shaking at 200 rpm at 37°C for 3 h, or until OD_600_ reaches 2.0. Cells were pelleted at 8000 × *g* for 5 min at room temperature and the pellet was resuspended in 200 µl lysis buffer [50 mM Tris, pH 8.0, 10 mM ethylenediaminetetraacetic acid (EDTA), 1% Triton X-100, 0.5 mg/ml RNase A, 20 mg/ml lysozyme] and incubated at 37°C for 30 min. Twenty microliters of 10% sodium dodecyl sulfate (SDS) and 20 µl protease K (10 mg/ml in TE buffer with 10% glycerol) were added and incubated at 55°C for 30 min. Five hundred microliters of PB buffer (5 M GuHCl, 30% isopropanol) was added and the entire sample volume was loaded onto a Qiagen QIAprep Spin Miniprep column and centrifuged at 13 000 × *g* for 1 min, followed by another 500 µl buffer wash. Seven hundred fifty microliters of PE buffer (10 mM Tris, pH 7.5, 80% ethanol) was added and centrifuged at 13 000 × *g* for 30 s, followed by one dry spin for 1 min. Columns were transferred to a new microcentrifuge tube and gDNA was eluted with 100 µl ddH_2_O at 13 000 × *g* for 1 min, reloaded and spun again. The gDNA concentration and purity was measured using a Nanodrop and samples were stored at −20°C.

### Spot titer sensitivity assays and MICs

*B. subtilis* strains were struck out onto the appropriate LB agar plates and incubated at 37°C overnight. A single colony per strain was inoculated in 2 ml LB media in 14 ml round bottom culture tubes, supplemented with spectinomycin when necessary. Cultures were incubated on a rolling rack at 37°C until the OD_600_ was 0.5–0.8. Cultures were normalized in 200 µl to an OD_600_ of 0.5 using 0.85% w/v sterile saline and were serially diluted in saline to 10^−5^. Plates were prepared the day of the assay with appropriate antibiotic and/or 100 µM isopropyl β-D-1-thiogalactopyranoside (IPTG) as indicated in figures. Five microliters of the serial dilutions were spotted onto prepared LB agar plates. Plates were grown at 37°C for 11 h or 25°C for 36 h. All spot titers were performed in biological triplicate and were imaged using the white light source in the Alpha Innotech MultiImage Light Cabinet, with exposure, brightness, and contrast edited in Adobe Photoshop. Minimum inhibitory concentration assays were performed as previously described with respective antibiotics [39].

### Total RNA isolation

Total RNA isolation was done for liquid chromatography coupled to tandem mass spectrometry (LC–MS/MS) and Nanopore procedures. For LC–MS/MS, 50 ml cultures were inoculated with a single colony of each appropriate strain. Antibiotics and IPTG were added to the *tlyA* complement cultures. Strains were grown until they reached an OD_600_ of 0.6–0.8. Forty milliliters were then centrifuged at max speed of tabletop centrifuge for 15 min. Pellets were then resuspended in 5.6 ml NE (0.1 M NaCl, 0.05 M EDTA) and incubated for 5 min at 37°C. Following, 300 µl of freshly made lysozyme (40 mg/ml in water) was added to each tube and incubated at 37°C for 15 min. Following incubation, 700 µl 10% sarkosyl was added and incubated on ice for 5 min. Lysates then underwent phenol:chloroform extraction by adding one volume of phenol:chloroform:iso-amyl alcohol (125:24:1) (Sigma P1944), shaking and vortexing, and spinning down tabletop centrifuge max speed for 30 min at 4°C. The aqueous phase was pipetted off the top and underwent an additional phenol extraction, followed by a final chloroform extraction. To precipitate the nucleic acids, 3× volumes 100% ethanol was added with 0.1× volume of 3M NaOAc to the extraction, inverted to mix, and then placed at −20°C for at least 2 h up to overnight. Following precipitation, nucleic acids were pelleted at max speed for 10 min at 4°C in tabletop centrifuge, washed twice with 70% ethanol, and dried at RT for ~10 min. The pellet was then resuspended in 5 ml water and precipitated again by mixing with 3× volumes ethanol and ½ volume 7.5M ammonium acetate and placing at −20°C for at least 2 h up to overnight. Following the second precipitation, nucleic acids were pelleted and washed again as previously described. The pellet was then resuspended in 400 µl water, transferred to a microcentrifuge tube and incubated with DNase I (Roche 04716728001) according to manufacturer’s instructions at 37°C. The reaction was quenched after 30 min with one phenol:chloroform:iso-amyl alcohol extraction and one chloroform extraction. RNA was precipitated at −20°C overnight with the addition of 3× ethanol and 0.1× NaOAc. Following, RNA was pelleted, washed with 70% ethanol, and dried as described above and resuspended in 80 µl water. Concentrations of 1:20 diluted product was determined by nanodrop for approximate or qubit (Broad Range RNA kit) for precise measurement. RNA quality was checked using bleach gel agarose electrophoresis [40]. Total RNA was then aliquoted and stored at −80°C for long-term storage. Total RNA extraction for Nanopore was performed in the same manner but was scaled down in volume starting with an initial 5 ml culture. Further for these isolations there was no additional phenol extraction upon initial clean up and the second pelleting, washing, and precipitation was abandoned as higher purity was not as necessary for these applications. For LC–MS/MS applications 23S and 16S rRNAs were further isolated using 1% agarose 0.5×Tris-borate-EDTA (TBE) gel electrophoresis and the Zymoclean Gel RNA Recovery kit (Zymo R1011) according to manufacturer’s instructions.

### Quantitative ribonucleoside LC–MS/MS

The ribonucleoside modification landscape was quantitatively assessed using a highly multiplexed targeted ribonucleoside LC–MS/MS assay as previously described [41]. Briefly, purified rRNA (150 ng) was enzymatically degraded to monoribonucleosides using a two-step digestion. The RNA was first hydrolyzed to ribonucleotide monophosphates using 300 U nuclease P1 (NEB, 100 000 U/ml) per μg of RNA overnight at 37°C in 100 mM ammonium acetate (pH 5.5) and 100 μM ZnSO4. The resulting ribonucleotides were dephosphorylated using 50 U bacterial alkaline phosphatase (Invitrogen, 150 U/µl) per μg of RNA for 4 hr at 37°C in 100 mM ammonium bicarbonate (pH 8.1) and 100 μM ZnSO4. Prior to the digestion, the enzymes were buffer exchanged into the respective digestion buffers using a BioRad Micro Bio-Spin 6 size exclusion column to remove glycerol and other ion suppressing contaminates. The resulting monoribonucleoside mixture was lyophilized and resuspended in 10 µl 40 nM 15N4-inosine as an internal standard. Following, the ribonucleosides were separated by reversed phase chromatography and quantified using multiple reaction monitoring on a triple quadrupole mass spectrometer [41].

### Nanopore

Ribosomal 23S or 16S RNA were pulled down from total RNA of either WT or ∆*tlyA* strains using custom-ordered reverse transcription adapters as described in the Oxford Nanopore Technologies Direct RNA Sequencing Kit manual (SQK-RNA002). Samples were then generated according to kit instructions and ran with a R9.41 flow cell (FLO-MIN106) using ONT’s MinION Mk1B technology. Two biological replicates were used for WT and ∆*tlyA* for each of the 23S and 16S rRNAs. Reads from WT and ∆*tlyA* were then compared using the Nanocompore pipeline according to its online manual to indicate modification location by analyzing differences in electrical current peaks and dwell time [42].

### Site-directed Mutagenesis

*B. subtilis* and *M. tuberculosis* TlyA alignment was done with web service alignment Tcoffee using default settings and visualized using Jalview Version 2. Site-directed mutagenesis was carried out using the efficient one-step single-site plasmid mutagenesis protocol, as previously described [43]. The DNA template used for amplification was pPB41, containing a wild-type copy of *tlyA* under the IPTG-inducible P_hyperspank_ promoter. Four catalytic mutants were constructed at residues K69, D155, K183, and E239 using the primers described in Supplementary Table S3. Three SAM-binding mutants were created at residues V63, G90, G94 using primers described in Supplementary Table S3. Modeling to determine relevant residues was done in Pymol using an alpha fold model of *B. subtilis* TlyA with SAM aligned from its position bound in methyltransferase HhaI (PDB code 2HMY). Plasmids were used to transform competent MC1061 and mini-prepped according to manufacturer’s instructions (Qiagen 27104). All constructs were Sanger sequenced to ensure *tlyA* was correctly mutated. Plasmids were used to transform ∆*tlyA*.

### Western blot

To ensure that the functionally mutated copies of TlyA were properly expressed, we conducted western blots using a custom polyclonal antiserum against TlyA. One colony was inoculated per strain in 25 ml LB media and was grown at 37°C for ~2 h until reaching an OD_600_ of ~0.5–0.8. Strains containing *tlyA* under the P_hyperspank_ promoter were either left uninduced or induced with 100 µM IPTG once at an OD_600_ between ~0.15–0.25 as noted in the figure. Cells were spun down and resuspended in 1 ml of lysis buffer (20 mM Tris, pH 8.0, 4M NaCl, 1M dithiothreitol (DTT), 0.5M EDTA, 10% SDS, urea, and water) and further lysed by sonication 60 Hz for 1 min total (10 s on/10 s off). Crude cell extracts were boiled for 5 min, mixed with 2× SDS loading dye, and applied to an SDS-polyacrylamide gel electrophoresis. Following, proteins were transferred to Cytiva Amersham nitrocellulose membranes using the Bio-Rad Trans-blot Turbo System set for using the system’s setting mixed weight protein transfer. Following transfer, membranes were incubated in 5% milk for blocking, followed by incubation with a 1/500 dilution of anti-TlyA (polyclonal antibody from exsanguination bleed of immunized MI-1947 rabbit by Covance) antibody or a 1/250 dilution anti-Myc [Abcam (9E10) ab32 lot #1117024-5] antibody for 1 h at room temperature. The membrane was then washed four times with TBS-T and incubated with fluorescent tagged LICOR 680 anti-Rabbit secondary antibody (Fisher NC9030093) or LICOR 800 anti-Mouse (Fisher NC9401841) for 1 h at room temperature. Then, the membrane was rinsed four times with TBS-T and imaged with a Licor Odessey Fc Imager.

### Growth curve of catalytic mutant

*B. subtilis* strains were struck out onto the individual LB agar plates supplemented with spectinomycin agar when needed and incubated at 37°C overnight. Strains were plate washed and standardized to a starting OD_600_ of 0.02 and inoculated in 12.5 ml of LB media in 125 ml Erlenmeyer flasks. Cultures were grown shaking at 200 rpm and 37°C for a total of 7 h, where the OD_600_ measurement was taken every 20 min. Since the overexpression of TlyA-K183A appeared to be catalytically lethal, IPTG was added at the beginning of the exponential phase (OD_600_ = 0.2). Growth measurements were taken in biological triplicate and were analyzed and graphically displayed using R.

### Sucrose gradient ultracentrifugation

Ribosomal subunit analysis was done by sucrose gradient ultracentrifugation as previously described [26]. *B. subtilis* cultures of 150 ml were grown up at 37°C or 25°C to an OD_600_ of 0.5–0.6. Cultures were pelleted via centrifugation at 4500 × *g* for 10 min, and each pellet was resuspended in 150 µl buffer G (20 mM Tri–HCl, pH 7.5, 200 mM NH_4_Cl, 6 mM β-mercaptoethanol) with either associating (10 mM) or dissociating (1 mM) Mg (OAc)_2_, 1 µl 50 mg/ml lysozyme, and 1× protease inhibitors (Pierce, EDTA-free). Resuspended cells were then lysed by sonication on ice. Following, lysates were DNase I (Roche 04716728001) treated for 10 min on ice following manufacturer’s instructions. Lysate was cleared by centrifugation in a tabletop microcentrifuge for 30 min at 16 000 × *g* and 4°C. Approximately 15 A_260_ units were added to the top of each sucrose gradient [10%–40% (w/v) in associating or dissociating buffer conditions] and were ultracentrifuged in a Thermo TH-641 rotor at 25 000 rpm for 15 hr at 4°C. Gradients were fractionated by hand into UV translucent 96-well plates. Each fraction was diluted 1:10 and the dilutions were measured for A_260_ by Tecan Infinite M100 or Tecan Spark spectrophotometers. Three biological replicates were used to quantify mean area under the curve for every strain and subunit. Subunit areas were separated based on the visualization of their peaks. Area under the curve was quantified using the Simpson’s rule method and normalized by dividing each subunit’s area by the total area of all three peaks. Significance was determined using an ANOVA with post hoc analysis using the Bonferroni method.

## Results

### ∆*tlyA* causes growth sensitivities and antibiotic resistance

**Figure 1.**
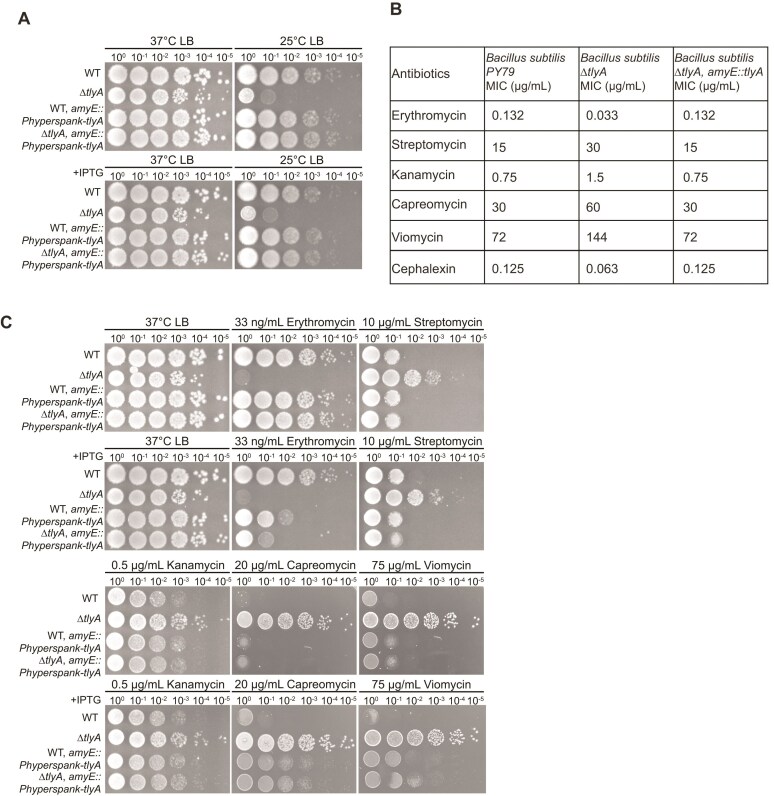
Spot-titer growth assays show cells with *∆tlyA* are sensitive to cold and erythromycin while resistant to aminoglycosides. Shown are spot-titer dilutions of the indicated strains plated on media under the conditions listed. (**A**) Tests cold sensitivity at 25°C. (**B**) Minimum inhibitory concentration (MIC) assays show ∆*tlyA* sensitivity or resistance to the antibiotics shown. (**C**) Cells were plated in the presence of the macrolide erythromycin, aminoglycosides kanamycin and streptomycin, and cyclic peptides capreomycin and viomycin. In each case *∆tlyA* is complemented using leak expression from the P_hyperspank_ promoter from an ectopic locus.

To determine the importance of TlyA in the cell and understand its function, the *tlyA* gene was deleted in the WT (PY79) background leaving only a *loxP* scar (see the “Materials and methods” section). Growth of ∆*tlyA* cells was then compared to WT using spot titer assays. Ribosome assembly defects have been shown to be exacerbated by cold temperatures [44]. Therefore, we compared growth at 37°C and 25°C (Fig. 1A). We noticed a strong growth sensitivity to cold for the ∆*tlyA* strain initially indicating its function could be related to the ribosome. We normally grow *B. subtilis* at 30°C, but because of the cold sensitivity all experiments were performed at 37°C where WT and ∆*tlyA* exhibit more similar growth. In the spot titer assay when grown at 25°C, the cold sensitivity was shown to be due solely to the loss of TlyA (Fig. 1A). Full complementation was observed in ∆*tlyA* when *tlyA* was expressed from the ectopic locus *amyE* with low expression from the uninduced promoter P_hyperspank_ or overexpression with β-D-1-thiogalactopyranoside (IPTG) (Fig. 1A). Overexpression of *tlyA* in a WT background did not cause a visible difference in growth compared to the WT control and complement in the deletion strain (Fig. 1A).

To determine whether methylation from *tlyA* resulted in altered sensitivity to ribosome-targeting antibiotics, we tested growth on plates with a variety of ribosome-targeting antibiotics including erythromycin, a macrolide targeting 23S rRNA near the peptide exit tunnel, and streptomycin and kanamycin, aminoglycosides targeting 16S rRNA near the ribosomal decoding site [45, 46] (Fig. 1B and C). Spot plate and MIC results show growth sensitivity to erythromycin and resistance to both aminoglycosides suggesting the modification on the 16S rRNA prevents the binding of these antibiotics to the ribosome. Further, we show that *B. subtilis* ∆*tlyA* shows resistance to cyclic peptide antibiotics capreomycin and viomycin, which bind the interface between the 30S and 50S subunits, which are second line antibiotics used to treat *M. tuberculosis* infections [35] (Fig. 1B and C). As a control, we used the cell wall antibiotic cephalexin in which ∆*tlyA* cells show slight sensitivity most likely due to their slower overall growth or because effects on translation in ∆*tlyA* will affect downstream cellular processes including cell wall synthesis (Fig. 1B and C). The *B. subtilis* ∆*tlyA* results differ from those found with loss of TlyA in *M. tuberculosis* where ∆*tlyA*_Mtb_ shows resistance to capreomycin and viomycin but does not show cross-resistance with aminoglycosides [28]. Interestingly, the overexpression of *tlyA* with the addition of IPTG only partially rescues the growth defect when challenged with erythromycin. This result suggests that excess TlyA is detrimental to cell physiology when challenged with some antibiotics. One possibility is that TlyA expressed at low native level does not modify every ribosome, and disrupting this balance by TlyA overexpression increases the proportion of modified ribosomes causing impaired growth when cells are challenged with ribosome-targeting antibiotics. As shown and described in greater detail below using western blots, native expression of TlyA is similar to uninduced expression from an IPTG-regulated promoter (Fig. 6A). This result explains how uninduced expression levels of TlyA can fully complement the erythromycin sensitivity. Taken together, the phenotypic growth assay results demonstrate effects on antibiotic efficacy and further suggest a functional role for TlyA in ribosome assembly.

### *Bacillus subtilis tlyA* forms 2′-O-methylcytidine modifications at 23S C1949 and 16S C1417 rRNAs

**Figure 2.**
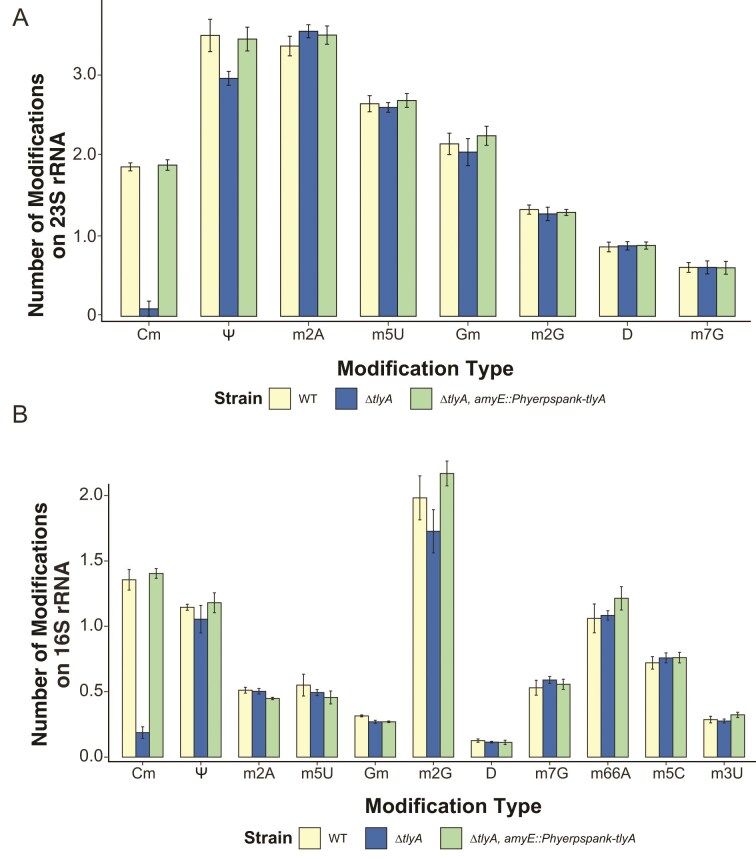
2′-O-methylcytidine modifications are absent from the 23S and 16S rRNA in *∆tlyA*. LC–MS/MS was used to identify rRNA modifications present in WT, *∆tlyA*, and the complementing control strain. (**A**) Shows modifications detected in 23S rRNA. (**B**) Shows modifications detected in the 16S rRNA. The modifications detected are as shown N^2^-methylguanosine (m^2^G), 2′-O-methylcytidine (Cm), pseudouridine (Ψ), C2-methyladenosine (m_2_A), 5-methylcytosine (m^5^C), 5-methyluridine (m^5^U), N7-methylguanosine (m^7^G), C2-methyladenosine (m^2^A), 2′-O-methylguanosine (Gm), 3-methyluridine (m^3^U).

Cold sensitivity and resistance to ribosome targeting antibiotics are consistent with methylation from TlyA impacting ribosome function. Therefore, we sought to identify the modifications conferred by TlyA on the 23S or 16S rRNA in *B. subtilis*. To do this, rRNAs were isolated and analyzed by LC–MS/MS [41] across three strains WT, ∆*tlyA*, and ∆*tlyA, amyE::*P_hyperspank_*-tlyA* as a complement control. The levels of 50 modifications were simultaneously quantified, including all modification types known to be conferred by *E. coli* rRNA methyltransferases [17]. Compared to WT, ∆*tlyA* showed a significant decrease in levels of Cm modifications on both the 23S and 16S rRNA (Fig. 2A and B, and Supplementary Tables S4 and S5). Ectopic chromosomal expression of *tlyA* from the IPTG-inducible P_hyperspank_ promoter in the ∆*tlyA* background restored Cm modification of the 23S and 16S rRNA. Our findings provide further support for a recent report showing that *B. subtilis* TlyA confers Cm modifications on the 23S and 16S rRNA [23]. We detected very low levels of five further varieties of nucleoside methylations in the 23S and eight in the 16S not previously identified (Fig. 2 and Supplementary Tables S4 and S5). We note that these observations are unlikely to arise from tRNA contamination as none of the common tRNA specific modifications (i.e. t^6^A, i^6^A) are detected. Instead, these modifications most likely originate from trace levels of contaminating rRNAs and are detected due to the highly sensitive nature of the triple quadrupole mass spectrometry assay employed here. However, the identification of the modification by TlyA remains clear as Cm modifications are the only modification significantly decreased in ∆*tlyA* and return to WT levels in the complementation strain.

**Figure 3.**
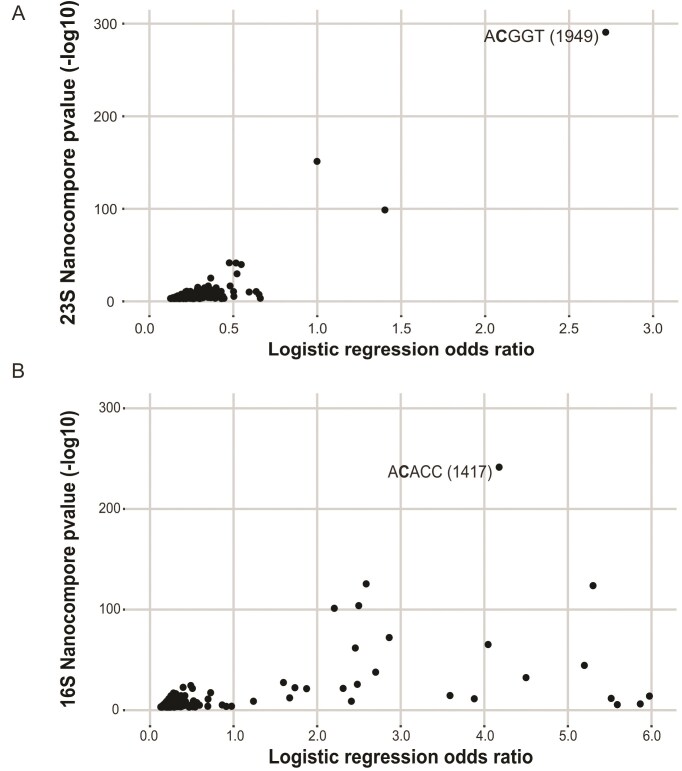
Nanopore sequencing shows modifications at C1949 in the 23S and C1417 of the 16S rRNA. Scatterplots show results of Nanocompore analyses comparing WT and *∆tlyA* reads from (**A**) 23S and (**B**) 16S reads. The plots show the absolute value of the Nanocompore logistic regression odd ratio (GMM logit method) on the *x*-axis plotted against the -log_10_ (*P*-value). Each point represents a *P*-value for a specific *k*-mer for *k-*mers with *P*-values <.001.

Given that *B. subtilis* TlyA is responsible for Cm modifications on the 23S and 16S rRNA, we sought to determine their locations within the rRNA sequences using Oxford Nanopore Technologies direct RNA sequencing. The Nanopore device pulls 5-nucleotide sections or *k*-mers through its pore outputting unique ionic currents and dwell times [42]. Outputs differ for modified bases when compared to nonmodified bases, enabling the detection of a single differentially modified base in WT and the methyltransferase deletion strain. Comparison between WT and ∆*tlyA* signal intensities and dwell times by Nanocompore analysis is represented by plotting the logistic regression log odd ratios and *P*-values of individual *k*-mers to depict the likelihood of a modification between ∆*tlyA* or WT rRNAs [28, 42]. *K-*mers in the 23S and 16S rRNA with the lowest *P*-value and highest log odds ratio indicate the modified cytidine residues [42, 47] (Fig. 3A and B). These results show modifications present at C1949 in the 23S rRNA and C1417 in the 16S rRNA, which are positions equivalent to those modified by TlyA*_Mtb_* and confirm the locations recently identified for *B. subtilis* TlyA [23, 28] (Fig. 3A and B). Peaks generated from comparing the signal intensities and dwell times between WT and ∆*tlyA* with logistic regression log odds ratio and Kolmogorov–Smirnov tests were also generated (Supplementary Fig. S1). Together our LC–MS/MS data, Nanopore data, and a previous publication [23] enable us to conclude that TlyA is necessary for the formation of Cm modifications on 23S C1949 and 16S C1417 in *B. subtilis*.

### Loss of *tlyA* causes an accumulation of the large subunit in associative conditions

**Figure 4.**
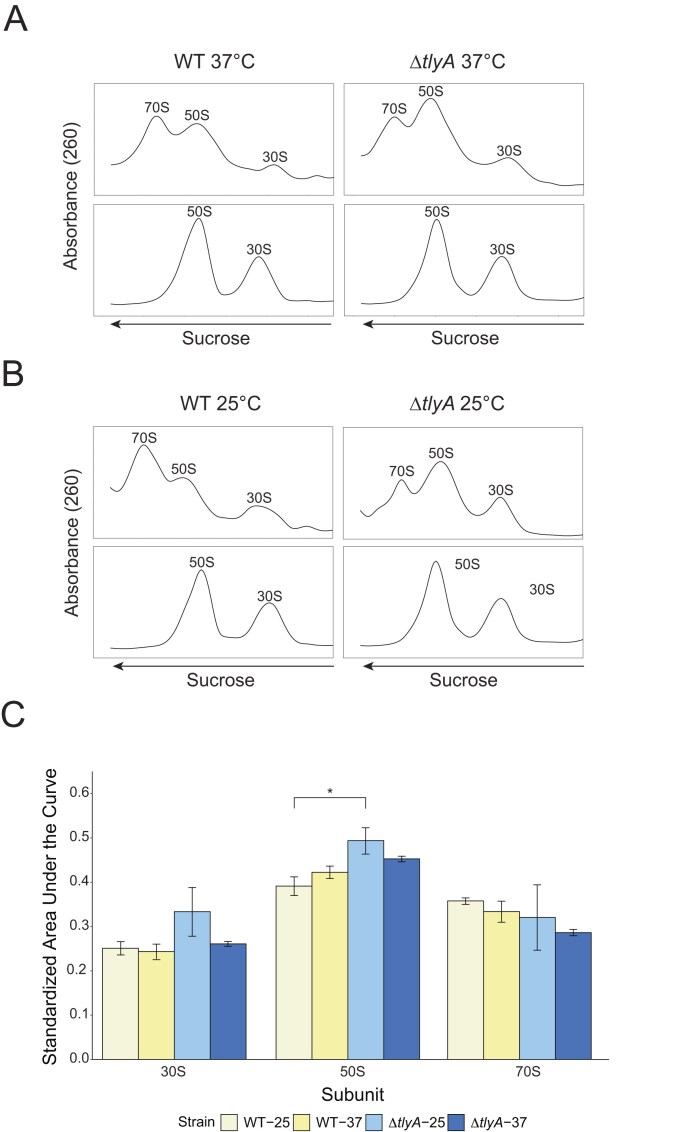
70S ribosome assembly is reduced in *∆tlyA* cells. Fractions from sucrose gradient ultracentrifugation in associating (top panels) or dissociating (bottom panels) buffers for WT and *∆tlyA* cells grown at (**A**) 37°C or (**B**) 25°C. (**C**) Associating sucrose gradient quantification of mean area under the curve for each subunit. Black bars represent standard error, and asterisk denotes significance (*P <.05).

Because ∆*tlyA* cells showed a growth defect at 25°C, we investigated the possible contribution of *tlyA* to ribosome assembly. To test ribosome assembly, we used sucrose gradient ultracentrifugation to quantify the formation of ribosomal subunits to determine whether ribosome assembly defects resulted from the absence of TlyA. We grew WT and ∆*tlyA* cultures at both 37°C and 25°C and then performed lysis and subsequent sample preparation in associating conditions where the ribosomal subunits come together to form the 70S mature ribosome or dissociating conditions where they remain as individual 50S and 30S subunits. Results from cultures grown at 37°C show no changes in dissociating conditions but do show a decrease in mature 70S formation in associating conditions (Fig. 4A). This difference is further exacerbated by cold growth conditions at 25°C, where the mature 70S peak is reduced in ∆*tlyA* compared to WT and there is a significantly higher level of 50S in ∆*tlyA* compared to WT (Fig. 4B and C). We also observe a small peak present in the ∆*tlyA* dissociating gradients at 25°C, which may be due to the presence of a 50S intermediate causing the significantly higher levels of 50S and reduced 70S in associating conditions. With these results, we conclude that Cm modifications from TlyA are necessary for proper subunit association to form a mature 70S ribosome.

### *tlyA_Mtb_* does not complement *B. subtilis* ∆*tlyA*

**Figure 5.**
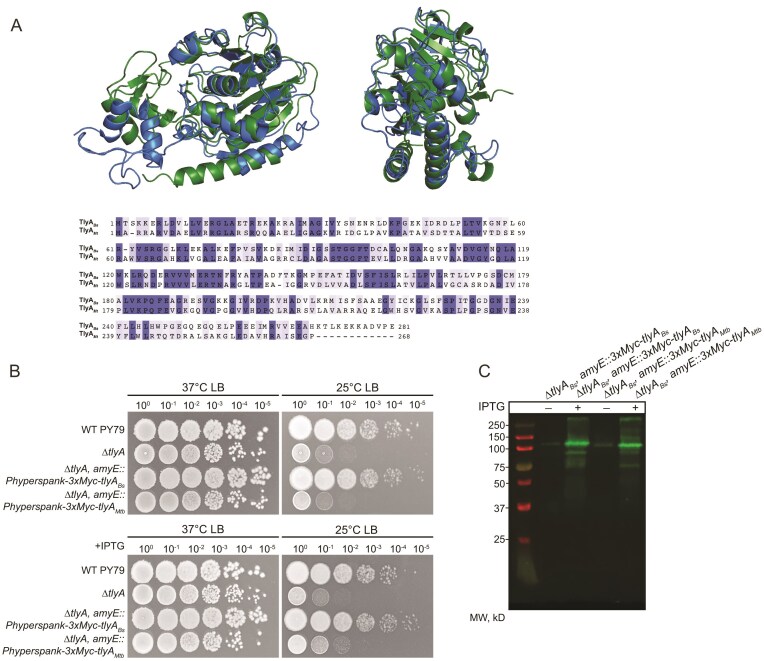
TlyA*_Mtb_* does not complement *B. subtilis ∆tlyA*. (**A**) AlphaFold 2.0 rendition of *B. subtilis* TlyA (green) shows similarity to the structure of TlyA*_Mtb_* (blue—PDB code 7S0S) from two views. Protein sequence alignment shows identical amino acids (purple) and similar amino acids (light purple) with 39% sequence identity and 54% sequence similarity shared between the two proteins. (**B**) Expression of *tlyA_Mtb_* from the *amyE* ectopic locus using leak expression from an IPTG-inducible promoter fails to complement *∆tlyA*. (**C**) Western blot displays expression of both 3xMyc tagged TlyA constructs from *B. subtilis* and *M. tuberculosis* from ectopic locus *amyE*. Overexpression is induced by 100 µM IPTG.

Since *B. subtilis* TlyA modifies the same rRNA positions compared to *tlyA_Mtb_* [28], we asked whether the *tlyA_Mtb_* could complement ∆*tlyA* in *B. subtilis*. Though these enzymes modify the same sequence contexts in their respective species, the enzymes share only 39% sequence identity and 54% sequence similarity and ∆*tlyA_Bs_* shows cross resistance to aminoglycoside antibiotics while ∆*tlyA_Mtb_* does not [28] (Fig. 5A). Further, we generated an AlphaFold model of TlyA*_Bs_* in green superimposed on the structure of TlyA*_Mtb_* in blue (PDB code 7S0S), revealing many shared interior α–helices and an overall RMSD of 1.572 but visible differences in their overall structures [48] (Fig. 5A). To determine whether *tlyA_Mtb_* can complement *B. subtilis tlyA* (*tlyA_Bs_*), we ectopically expressed a *B. subtilis* codon optimized *tlyA_Mtb_* from *amyE* in *B. subtilis* ∆*tlyA* cells with an N-terminal 3xMyc tag (see the “Materials and methods” section). As before, we show that ∆*tlyA_Bs_* confers reduced growth at 25°C. Expression of *tlyA_Mtb_* failed to complement growth of ∆*tlyA_Bs_* using uninduced expression or overexpression (Fig. 5B). As a control for protein expression, we show that 3xMyc-TlyA*_Mtb_* and 3xMyc-TlyA_Bs_ enzymes accumulate to the same amount *in vivo* (Fig. 5C). Therefore, the lack of complementation may be caused by structural differences between *B. subtilis* and *Mtb* TlyA or differences in how these enzymes bind to the ribosome to recognize their cognate substrate. One such difference may be present in the linker region between the N- and C-terminal domains. It has been noted that the *Mtb* TlyA linker is important for function and contains four amino acids ^60^RAWV^63^ compared to the *Bs* TlyA linker that contains three as ^61^RYV^63^ [49]. Overall structural differences are also present between the NTDs that direct substrate interaction as these align with an RMSD greater than 3.0, while the C-terminal domains (CTDs) align with an RMSD of 1.348 displaying higher structural conservation for the CTD. The presence of Cm modifications at homologous sites on the 23S and 16S rRNA in *B. subtilis* and *Mtb* suggest conservation between Firmicutes, Actinomycetota, and other TlyA containing bacteria. However, we find that *Mtb* TlyA is not interchangeable with *B. subtilis* TlyA. Therefore, although *B. subtilis* and *Mtb tlyA* modify equivalent positions in the 16S and 23S, the gene deletions yield different phenotypes and *tlyA_Mtb_* is unable to complement ∆*tlyA_Bs_*_._ These experiments suggest important differences between TlyA from *Mtb* and *B. subtilis*.

### Residues K69, D155, and K183 are required for catalytic activity of TlyA *in vivo*

**Figure 6.**
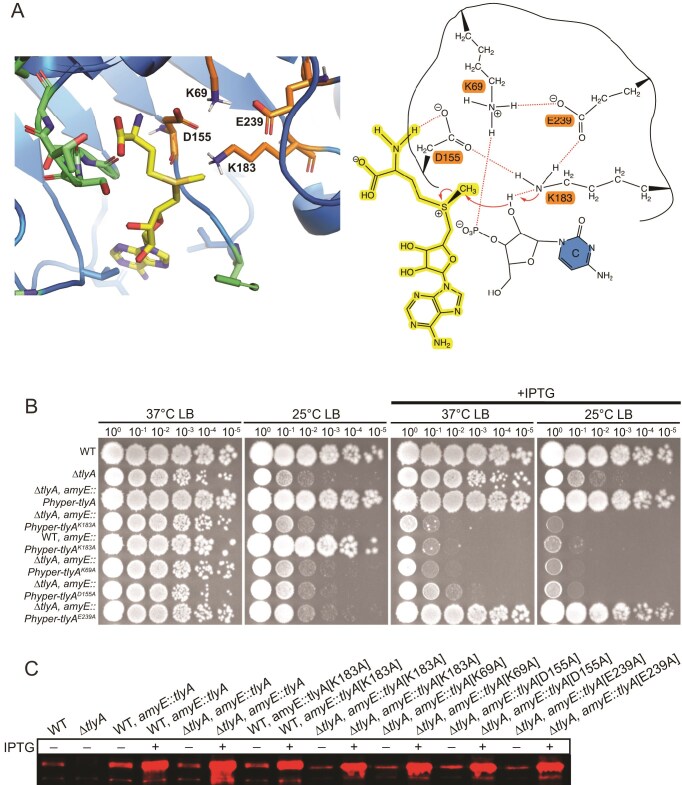
Residues K99, D155, K183, and E239 are important for function of TlyA *in vivo*. (**A**) PyMOL model showing SAM cofactor positioning in the catalytic tetrad of TlyA. Catalytic residues are colored in orange with the supporting SAM-binding residues shown in green. ChemDraw illustration of the proposed methyl transfer reaction of TlyA with the SAM cofactor shown in yellow, catalytic residues in orange, and the cytidine bearing the 2′-hydroxyl substrate in blue. (**B**) Spot titer assays showing failure of TlyA variants to complement the growth defect of ***∆****tlyA* expressed from the ectopic locus *amyE* from an IPTG inducible promoter. (**C**) Western blot of TlyA shows mutants are expressed and accumulate to WT levels with and without addition of IPTG.

To further understand TlyA function, we investigated putative catalytic residues. The catalytic site of *B. subtilis* TlyA was proposed based on homology to the *E. coli* heat-shock methyltransferase RrmJ [50]. To determine residues required for *in vivo* function of *B. subtilis* TlyA, we created four mutants using site-directed mutagenesis at residues *K69, D155, K183*, and *E239* that are predicted to be important for catalysis [50]. Western blotting of TlyA variants showed similar expression levels compared to WT TlyA expression using antiserum raised against TlyA*_Bs_* (Fig. 6C and Supplementary Fig. S2). When expressed from an ectopic locus in a *∆tlyA* background, the *K183A, K69A*, and *D155A* mutants were unable to rescue the phenotype of ∆*tlyA* under cold growth conditions, regardless of expression level (Fig. 6B). The mutant *E239A* was only able to rescue the phenotype of ∆*tlyA* when induced with IPTG, indicating that only overexpression of this variant was able to compensate for ∆*tlyA*.

As shown in Fig. 6A, the glutamate at position 239 is the most removed from the catalytic tetrad and could explain how only overexpression allows for complementation of the deletion. Based on the model, the K-D-K residues of the catalytic tetrad are directly stabilizing or acting on the cytidine or SAM cofactor (Fig. 6A). We generated a PyMOL model that depicts the SAM cofactor positioned in the active site of TlyA, where the K183 is oriented for the deprotonation of the 2′-hydroxyl and nucleophilic attack to transfer the methyl group from SAM to the cytidine. When growing our strains, we noticed that overexpression of the *K183A* mutant in ∆*tlyA* halted cell growth in LB media at 37°C. To determine whether *K183A* is dominant negative to WT, we expressed *tlyA^K183A^* from the P_hyperspank_ promoter in a WT background. Growth curves show that induced overexpression of *tlyA^K183A^* drastically reduces cell growth during exponential phase in a WT background, suggesting this variant is indeed dominant to the WT protein (Supplementary Fig. S3). Given the importance of the three residues that are predicted to work directly on the substrate and cofactor, we propose that TlyA functions with the K-D-K catalytic triad, similar to RrmJ, with the glutamate supporting catalytic activity [51, 52].

### Bacillus subtilis TlyA uses the GxSxG motif to bind SAM in vivo

**Figure 7.**
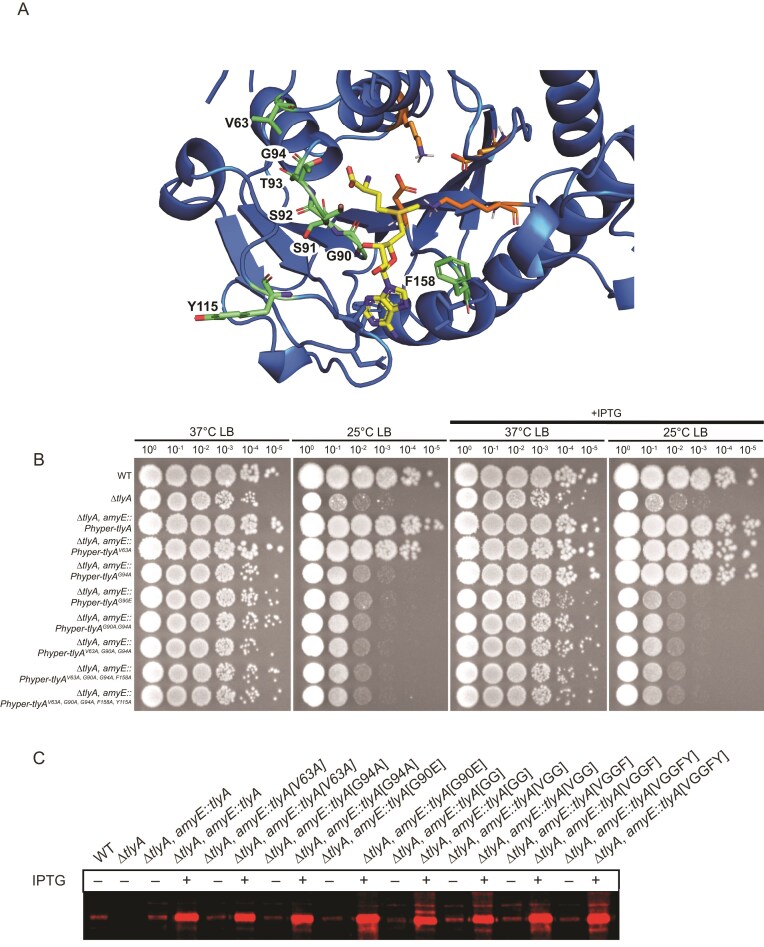
The GxSxG motif helps stabilize the SAM cofactor in the active site of TlyA *in vivo*. (**A**) AlphaFold 2.0 depiction of TlyA*_Bs_* (blue) with residues in green (G90–G94 and V63) used to help stabilize the binding of SAM (yellow) aligned from its position bound in methyltransferase HhaI (PDB code 2HMY). Aromatic residues Y115 and F158 may provide additional stabilization of SAM or the nucleoside substrate. (**B**) Spot-titer assay shows *tlyA* mutant complementation of ***∆****tlyA* growth to at 25°C to identify mutants that are important of SAM binding *in vivo* as judged by failed complementation. **(C)** Western blot shows SAM-binding mutants are expressed and accumulate to native TlyA levels in vivo. GG corresponds to *G90A, G94A* and *VGG* corresponds to *V63A, G90A*,and *G94A* all of which were expressed from *amyE* using the P_hyperspank_ promoter in a *∆tlyA* genetic background where indicated.

After determining residues important for *in vivo* catalytic function, we asked which residues could be important for TlyA to bind the SAM cofactor. Many rRNA methyltransferases contain Class I MTase structure with the GxGxG motif, widely recognized to bind SAM [51, 52]. *Mtb* and *B. subtilis* TlyA both contain ^90^GxSxG^94^, a variant of the GxGxG motif [49] (Fig. 7A). In addition to the GxSxG motif, TlyA*_Mtb_* also requires the novel motif ^60^RXWV^63^ in the N-terminal domain (NTD) to CTD linker [49]. The RXWV motif organizes the G×S×G motif with the W62 and V63 being the most important for SAM affinity [49]. The corresponding residues in *B. subtilis* TlyA, ^61^RYV^63^, show the conserved valine in the linker region. To determine whether these residues are important for TlyA function *in vivo*, we again utilized *tlyA* mutant complementation growth assays. Western blotting shows that all TlyA variants are expressed at similar levels to WT (Fig. 7C and Supplementary Fig. S2). Spot titer assays demonstrate that substitution of one glycine can be tolerated, but the substitution of both glycine residues results in the inability of ectopically expressed TlyA variants to rescue the ∆*tlyA* phenotype at 25°C (Fig. 7B). When just a single glycine was substituted for a negatively charged residue (Glu), TlyA was nonfunctional *in vivo*. Upon mutating V63 to alanine, no growth defects were observed, demonstrating that TlyA*_Bs_* does not require the same linker region as TlyA*_Mtb_* does during SAM binding (Fig. 7B). Together, our results support the conclusion that TlyA*_Bs_* binds SAM via its ^90^GxSxG^94^ motif *in vivo*. The linker region, with its conserved valine residue, is not required for SAM binding by *B. subtilis* TlyA *in vivo*, demonstrating yet another functional difference between *B. subtilis* and the *Mtb* TlyA enzymes.

## Discussion

In this study, we characterize the *tlyA* gene (previously *yqxC*), as a dual-substrate 16S and 23S 2′-O-methylcytidine (Cm) rRNA methyltransferase important for ribosome assembly in *B. subtilis*. Prior work has identified some of the genes encoding rRNA methyltransferases in *B. subtilis*; however, none have been shown to have distinct phenotypes that effect growth or ribosome assembly [24–27]. Hence, our study of TlyA and its distinct deletion phenotypes are uniquely important for understanding ribosome biogenesis, ribosome function, and antibiotic resistance in Gram-positive and other related bacteria.

The phenotypes of ∆*tlyA* we found are due to loss of 2′-O-methylcytidine modifications on the 23S and 16S rRNA and maintenance of all other methylation modifications as shown by our LC–MS/MS results. Our results generally match recent LC–MS/MS data for *B. subtilis* 16S and 23S rRNA that was published as our work was ongoing [23]. Some differences in modification numbers from our quantification are most likely due to the high sensitivity of the LC–MS/MS procedure detecting trace levels of the 23S or 16S rRNAs in the alternate isolates increasing the number of certain modifications that are present on both rRNAs. Utilizing Oxford Nanopore direct RNA sequencing, we confirmed the location of these modifications to be 23S C1949 and 16S C1417. In the structure of the ribosome, these modifications exist on Helix 69 of the 23S rRNA and helix 44 of the 16S rRNA. Helix 44 is part of the aminoacyl (A) site that accepts incoming tRNAs, and Helix 69 is located between the A and P sites and has been shown to be essential for decoding mRNA and bridging to helix 44 of the 16S creating a connection between the large and small subunits [53, 54]. Biochemically, the formation of Cm modifications by TlyA would be expected to reduce the reactivity of two different 2′-hydroxyl groups, which increases RNA stability and promotes correct conformation of the rRNA especially necessary in this functionally important area that provides an allosteric link between the large and small subunits.

Our spot plate analyses show cold sensitivity upon deletion of *tlyA*. This is most likely due to exacerbated ribosome assembly defects at 25°C as seen in our sucrose gradient results with an increase in the 50S subunit in associative conditions compared to WT. *Escherichia coli ∆rlmE* strains show a similar defect with an accumulation of a 45S large subunit intermediate due to incomplete processing by RNase III suggesting that the modifications conferred by TlyA are necessary for 23S processing in *B. subtilis* [55, 56]. Spot plates also indicated a unique difference between *B. subtilis* and *M. tuberculosis*. In *Mtb*, ∆*tlyA* cells are only resistant to the second line cyclic peptide antibiotics viomycin and capreomycin [28], whereas ∆*tlyA* in *B. subtilis* is resistant to these antibiotics as well as the aminoglycosides kanamycin and streptomycin. Both cyclic peptide and aminoglycosides bind at similar locations to the interface of helix 44 in the small subunit and Helix 69 in the large subunit corresponding to the locations of the methylations conferred by TlyA at C1949 in Helix 69 of the 23S rRNA and C1417 in helix 44 of the 16S rRNA [57, 58].

In *M. smegmatis*, TlyA conferred methylations have been shown to cause structural rearrangements at the Helix 69–helix 44 interface causing an open conformation that allows efficient binding of capreomycin [59]. Loss of these modifications has been shown to cause more compaction of the tip of Helix 69 creating a steric hindrance blocking the capreomycin binding pocket [59].We suggest that similar structural changes occur with TlyA loss in *B. subtilis*, and find it likely that additional small structural differences in the *B. subtilis* and *Mtb* ribosomes upon loss of these modifications causes ∆*tlyA*_Bs_ to be resistant to both aminoglycosides and cyclic peptide antibiotics. Though loss of these modifications leads to resistance to a variety of antibiotics, *B. subtilis* likely retains this enzyme due to its importance for efficient rRNA processing and ribosome maturation allowing for rapid growth. Small differences in the rRNA substrate structure may also be the reason we find *tlyA_Mtb_* does not complement ∆*tlyA_Bs_ in vivo*. Additionally, structural differences between these enzymes could be important for their ability to recognize and modify native rRNA prohibiting cross-species complementation and differences in native deletion phenotypes. Together these data demonstrate the distinct antibiotic resistance and ribosome assembly phenotypes in ∆*tlyA B. subtilis* and potentially uncovers differences in the ribosome structures upon loss of TlyA modifications.

To test functionally important residues, which could be unique to TlyA in *B. subtilis*, we mutated both potential catalytic and SAM binding residues in TlyA*_Bs_*. Like many other methyltransferases, TlyA*_Bs_* contains a common Class I MTase Rossmann-like methyltransferase fold that has been shown to use the catalytic tetrad of residues K38, D124, K164, and E199 in the methyl transfer reaction [50, 60]. We found residues K69, D155, and K183 are indispensable for the function of TlyA in *B. subtilis*; however, TlyA^E239A^ can only complement ∆*tlyA* when overexpressed from an ectopic locus. For SAM binding, *in vitro* assays demonstrate that TlyA*_Mtb_* utilizes the SAM binding motif I GxSxG and a novel RXWV tetrapeptide motif linking the NTD and CTD to hold SAM in the active site [48, 49]. TlyA*_Bs_* has residues G90, S91, S92, T93, and G94, comprising the GxSxG motif, and V63 in the linker region. We tested residues that are required for SAM binding in TlyA*_Mtb_* and are present in TlyA*_Bs_* to understand the degree of enzymatic conservation between orthologs. In TlyA*_Bs_*, we found that glycine residues in the SAM motif but not the valine linker are necessary for function *in vivo*. Our results illustrate that for *B. subtilis*, TlyA only requires the GxSxG motif *in vivo*, indicating that there are functional differences in TlyA rRNA methyltransferases across bacteria. This difference may be due to the linker being shorter in *B. subtilis* composed of only three amino acids instead of four as it is in *Mtb* causing differences in RNA recognition by positioning of the NTD and subsequent catalysis. The presence of this shortened linker may contribute to the inability of the *tlyA_Mtb_* to complement the *B. subtilis tlyA* deletion. However, our results are limited in their *in vivo* study because cell growth was used as an indirect measure of TlyA variant function. Defining the residues necessary for the *in vivo* function of TlyA*_Bs_* demonstrates that active site residues are similar between rRNA methyltransferases from Gram-positive and Gram-negative species, yet differences in residues required to bind SAM between TlyA*_Bs_* and TlyA*_Mtb_* highlight another enzymatic difference that may prevent the enzymes from functioning interchangeably.

Our work characterizes the first rRNA methyltransferase in *B. subtilis* that shows a distinct phenotype including antibiotic resistance and a ribosome assembly defect. An rRNA methyltransferase modifying both the 23S and 16S is not present in *E. coli* demonstrating an evolutionary difference between Gram-positive, diderm, and many Gram-negative bacteria. It will be important to learn whether other rRNA methyltransferases in *B. subtilis* have similar phenotypes and effects on ribosome assembly, or whether TlyA is the most critical rRNA methyltransferase for ribosome biogenesis.

## Supplementary Material

gkaf1531_Supplemental_Files

## Data Availability

All Nanopore sequencing data used to identify modification location sites are available at Sequence Read Archive by NCBI through accession number PRJNA1237866. All other resources are described.
