# Supplementary material for: TlyA is a 23S and 16S 2′-O-methylcytidine methyltransferase important for ribosome assembly in Bacillus subtilis
Source: Nucleic Acids Res. 2026 Jan 15;54(2):gkaf1531. doi: 10.1093/nar/gkaf1531 (PMC12805891; doi:10.1093/nar/gkaf1531)
Supplement: gkaf1531_Supplemental_Files [file gkaf1531_supplemental_files.zip › 4_Supplementary_Information_New_docx.pdf]

Supplementary Information

TlyA is a 23S and 16S 2′-O-methylcytidine methyltransferase important for ribosome assembly in *Bacillus subtilis*

Jennie L. Hibma[1]†, Lia M. Munson[1]†, Joshua D. Jones[2], Taylor M. Nye[1], Kristin S. Koutmou[2]*, Lyle A. Simmons[1]*

[1]Department of Molecular, Cellular, and Developmental Biology, University of Michigan, Ann Arbor, MI 48109; [2]Department of Chemistry, University of Michigan, Ann Arbor, Michigan 48109.

† These authors contributed equally

*LAS: Department of Molecular, Cellular, and Developmental Biology, University of Michigan, Ann Arbor, Michigan 48109-1055, United States. E-mail: lasimm@umich.edu

*KSK: Department of Chemistry, University of Michigan, University of Michigan, Ann Arbor, MI Michigan 48109-1055, United States. Electronic address: kkoutmou@umich.edu

Supplementary Table S1: Strains used in this work

**Strains**	**Genotype**	**Citation**
JLH005	WT PY79	
JLH010	Δ*tlyA*	This work
JLH018	Δ*tlyA, amyE::*$P_{hyperspank}$*-tlyA*	This work
JLH020	MC1061	
JLH022	WT, *amyE::*$P_{hyperspank}$*-tlyA*	This work
JLH155	Δ*tlyA, amyE::*$P_{hyperspank}$*-tlyA*[*K183A*]	This work
JLH056	Δ*tlyA, amyE::*$P_{hyperspank}$*-tlyA*[*V63A*]	This work
JLH162	Δ$tlyA_{Bs}$, *amyE::*$P_{hyperspank}$-$tlyA_{Mtb}$	This work
JLH164	Δ*tlyA, amyE::*$P_{hyperspank}$*-tlyA*[*K69A*]	This work
JLH165	Δ*tlyA, amyE::*$P_{hyperspank}$*-tlyA*[*D155A*]	This work
JLH166	Δ*tlyA, amyE::*$P_{hyperspank}$*-tlyA*[*E239A*]	This work
JLH167	WT, *amyE::*$P_{hyperspank}$*-tlyA*[*K183A*]	This work
JLH168	Δ*tlyA, amyE::*$P_{hyperspank}$*-tlyA*[*G94A*]	This work
JLH171	Δ*tlyA, amyE::*$P_{hyperspank}$*-tlyA*[*G90E*]	This work
JLH172	Δ*tlyA, amyE::*$P_{hyperspank}$*-tlyA*[*G90A+G94A*]	This work
JLH173	Δ*tlyA, amyE::*$P_{hyperspank}$*-tlyA*[*V63A+G90A+G94A*]	This work
JLH249	Δ*tlyA*, *amyE::*$P_{hyperspank}$-*3xMyc*-$tlyA_{Mtb}$	This work
JLH250	Δ*tlyA*, *amyE::*$P_{hyperspank}$-*3xMyc*-$tlyA_{Bs}$	This work

Supplementary Table S2: Plasmids used in this work

**Plasmid**	**Vector**	**Insert**	**Reference/Source**
pDR244	pDR243	Cre-recombinase	Bacillus Genetic Stock Center (ECE274)
pJLH004	pPB194[1]	*tlyA*	This study
pLMM005	pPB194	*tlyA*[*K183A*]	This study
pLMM006	pPB194	t*lyA*[*V63A*]	This study
pLMM014	pPB194	t*lyA*[*K69A*]	This study
pLMM015	pPB194	t*lyA*[*D155A*]	This study
pLMM016	pPB194	t*lyA*[*E239A*]	This study
pLMM017	pPB194	t*lyA*[*G94A*]	This study
pLMM018	pPB194	t*lyA*[*G90E*]	This study
pLMM019	pPB194	t*lyA*[*G90E+G94A*]	This study
pLMM020	pPB194	t*lyA*[*G90E+G94A+V63*]	This study
pJLH048	Twist	3XMyc-$TlyA_{Mtb}$	This study
pJLH049	pPB194	3XMyc-$TlyA_{Mtb}$	This study
pJLH050	pPB194	3XMyc-$TlyA_{Bs}$	This study

Supplementary Table S3: Primers used in this work: Complimentary regions are underlined. Mutated nucleotides are bolded for mutagenesis primers.

Primer	Sequence	Purpose
PEB3F	GCTAGCCGCATGCAAGCTAATTCG	for amplifying pPB194 or pDR110
PEB259	ATGTATACCTCCTTAGTCGACTAAGCTTAATTGTTATCCGCTCACAATTACACACATTATGCCACACCTTGTAGATA	for amplifying pPB194 or pDR110
JLH040	GCAGGCGAGAAAGGAGAG	ermR cassette forward
JLH040a	CGAGGCTCCTGTCACTGC	ermR cassette reverse
JLH068	TTTAGTT**GC**GCCTCAGTTTGAAGCGGGACGGGAATCCG	TlyA^{K183A} catalytic mutant forward
JLH068a	CAAACTGAGGC**GC**AACTAAAGCCATGCAGTCGCTGCC	TlyA^{K183A} catalytic mutant reverse
JLH069	AGGTATG**C**GAGCAGGGGCGGCTTAAAGCTCGAAAAAGCGTTG	TlyA^{V63A} SAM binding mutant forward
JLH069a	CCTGCTC**G**CATACCTCAGCGGGTTTCCTTTGACAGTTAACGGAAG	TlyA^{V63A} SAM binding mutant reverse
JLH077	GTGTGTAATTGTGAGCGGATAAC	pPB194 sequencing forward
JLH077a	CAAAATCGTCTCCCTCCGTTTG	pPB194 sequencing reverse
JLH101	GGCTTAGC**GC**TCGAAAAAGCGTTGAAGGAATTTCCC	TlyA^{K69A} catalytic mutant forward
JLH101A	TTTTCGAGC**GC**TAAGCCGCCCCTGCTCACATAC	TlyA^{K69A} catalytic mutant reverse
JLH102	ACAATTG**C**TGTGTCCTTTATTTCACTGCGGCTC	TlyA^{D155A} catalytic mutant forward
JLH102a	AAAGGACACA**G**CAATTGTGGCAAACTCCGGC	TlyA^{D155A} catalytic mutant reverse
JLH103	CGGAAATATTG**C**GTTTCTCCTTCATTTGCATTGGCCG	TlyA^{E239A} catalytic mutant forward
JLH103a	GGAGAAAC**G**CAATATTTCCGTCTCCTCCCGTGATTG	TlyA^{E239A} catalytic mutant reverse
JLH105	CACCG**C**CGGTTTTACGGACTGCGCTTTGCAAAATGG	TlyA^{G94A} SAM binding mutant forward
JLH105a	GTAAAACCG**G**CGGTGGAGGAGCCAATATCAATCATAATTTTATCTTTGAC	TlyA^{G94A} SAM binding mutant reverse
JLH106	GATATTG**AG**TCCTCCACCGGCGGTTTTACGGACTGCG	TlyA^{G90E} SAM binding mutant forward
JLH106a	GGTGGAGGA**CT**CAATATCAATCATAATTTTATCTTTGACAGAGACGGGAAATTCCTTC	TlyA^{G90E} SAM binding mutant reverse
JLH109	GATATTG**C**CTCCTCCACCGCCG**G**TTTTACGGACTGCG	TlyA$^{G90A+G94A}$ SAM binding mutant forward
JLH109a	CGTGGAGGAG**G**CAATATCAATCATAATTTTATCTTTGACAGAGACGGGAAATTCCTTC	TlyA$^{G90A+G94A}$ SAM binding mutant reverse
JLH110	CGCATGATCTCTTCTTCCG	upstream 817 bp of Phyperspank
JLH110a	CAATGGTTCAGATACGACGAC	downstream 752 bp of Phyperspank
JLH138	CTTAGTCGACTAAGGAGGTATACATATGGAACAAAAGTTAATCTCAGAGGAGG	N-terminal TlyA-Mtb into pPB41 under amyE
JLH138a	GAATTAGCTTGCATGCGGCTAGCTTATCAAGGCCCTTCGGAGATCGCTCGATG	N-terminal TlyA-Mtb into pPB41 under amyE
JLH141	CAGAAGAGGACCTCGGAGGCGGTAGCATGACGTCAAAGAAAGAACGA	insert yqxC under N-terminal 3xMyc in pPB41
JLH142a	CATCTAATCGTTCTTTCTTTGACGTCATGCTACCGCCTCCGAGGTCCTCTTCTG	vector for yqxC under N-terminal 3xMyc in pPB41
JLH147	CTCGGAGGCGGTAGCATGGCAAGACGAGCCAGAGTG	site directed mutagenesis to remove 3xMyc stop codon for TlyA
JLH147a	CCATGCTACCGCCTCCGAGGTCCTCTTCTGATATAAGTTTTTG	site directed mutagenesis to remove 3xMyc stop codon for TlyA

Supplementary Table S4: Modifications per rRNA molecule

23S

**Nucleoside**	**WT**	**Δ*tlyA***	**Δ*tlyA*, *amyE*:: *tlyA***
Pseudouridine	3.492	2.959	3.448
2-methyladenosine	3.361	3.545	3.497
5-methyluridine	2.644	2.598	2.684
2′-O-methylguanosine	2.144	2.042	2.245
2′-O-methylcytidine	1.858	0.094	1.881
N2-methylguanosine	1.327	1.274	1.292
Dihydrouridine	0.863	0.877	0.880
7-methylguanosine	0.608	0.609	0.604
N6,N6-dimethyladenosine	0.035	0.024	0.015
5-methylcytidine	0.033	0.034	0.015
1-methylguanosine	0.015	0.013	0.012
3-methyluridine	0.013	0.010	0.003
N6-methyladenosine	0.004	0.003	0.002
2′-O-methylinosine	0.002	0.002	0.003

16S

**Nucleoside**	**WT**	**Δ*tlyA***	**Δ*tlyA*, *amyE*:: *tlyA***
N2-methylguanosine	1.983	1.727	2.169
2′-O-methylcytidine	1.356	0.187	1.404
Pseudouridine	1.146	1.055	1.181
N6,N6-dimethyladenosine	1.061	1.084	1.214
5-methylcytidine	0.721	0.759	0.761
5-methyluridine	0.551	0.494	0.456
7-methylguanosine	0.530	0.590	0.557
2-methyladenosine	0.511	0.503	0.448
2′-O-methylguanosine	0.315	0.270	0.270
3-methyluridine	0.287	0.276	0.323
Dihydrouridine	0.126	0.115	0.112
1-methylguanosine	0.004	0.006	0.004
5-methoxyuridine	0.003	0.005	0.004
N6-methyladenosine	0.002	0.008	0.004
2′-O-methylinosine	0.001	0.001	0.002

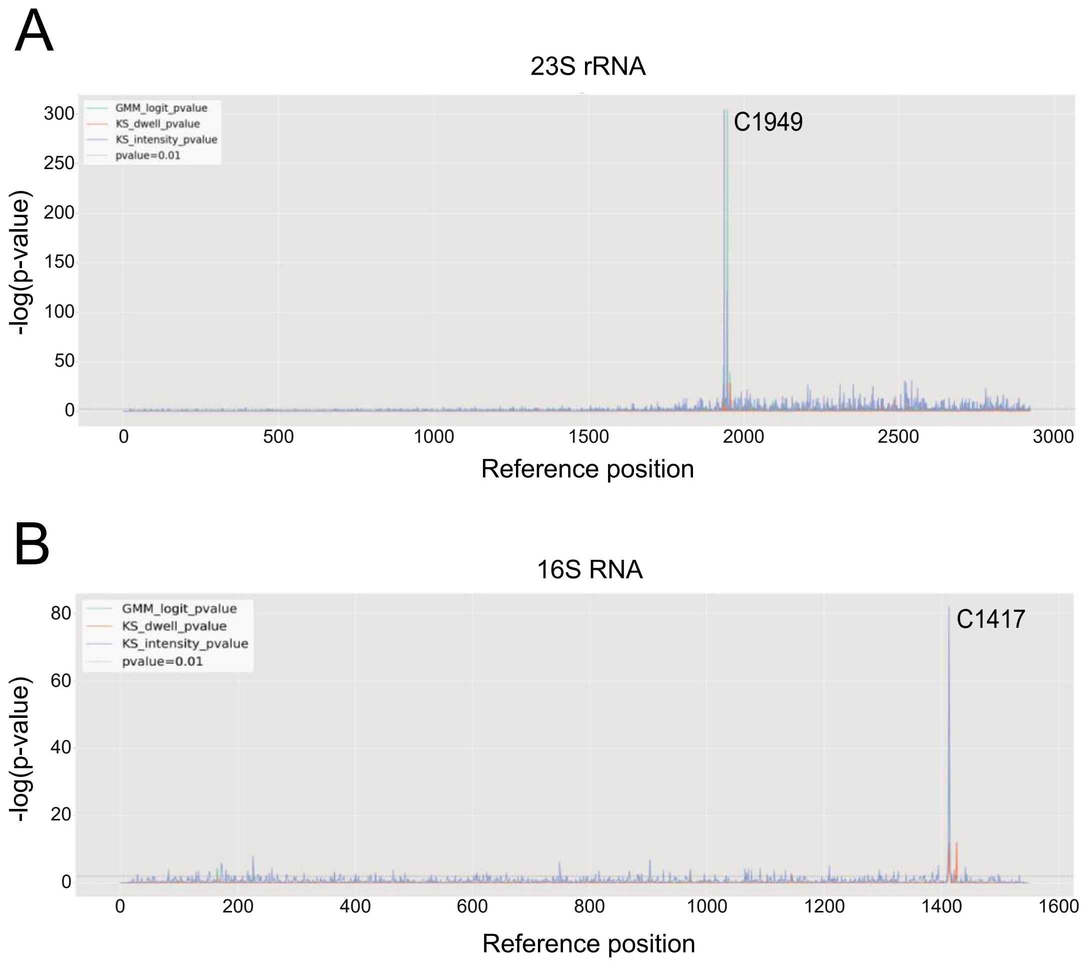

Supplementary Figure S1. Nanocompore analysis shows modified cytidine residue locations in the 23S at C1949 and in the 16S at position C1417. Shown are p-values from logistic regression log odds ratio (GMM) and Kolmolgorov-Smirnov tests for intensity value and dwell time by Nanocompore analysis for every reference position. The p-value peaks identify C1949 for the 23S **(A)** and C1417 for the 16S **(B)**.

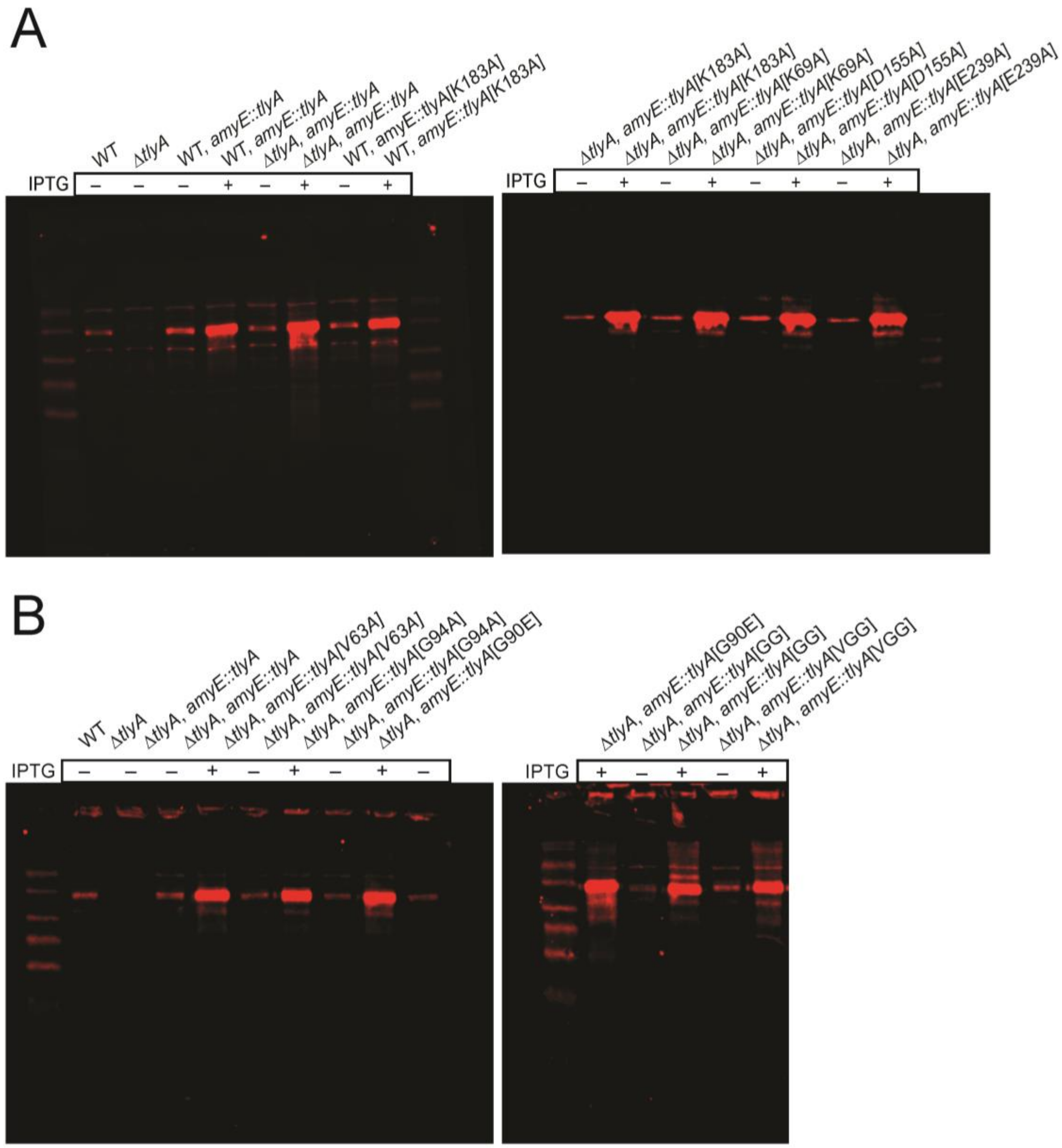

Supplementary Figure S2. Full Western blot shows TlyA expression in mutant strains is similar to WT. Western blot images show variant TlyA protein accumulation is similar to WT in whole cell lysates. Lysates from Δ*tlyA* is used as a negative control to show the major immunoreactive band corresponds to TlyA. **(A)** catalytic variant strains and **(B)** SAM binding mutant strains.

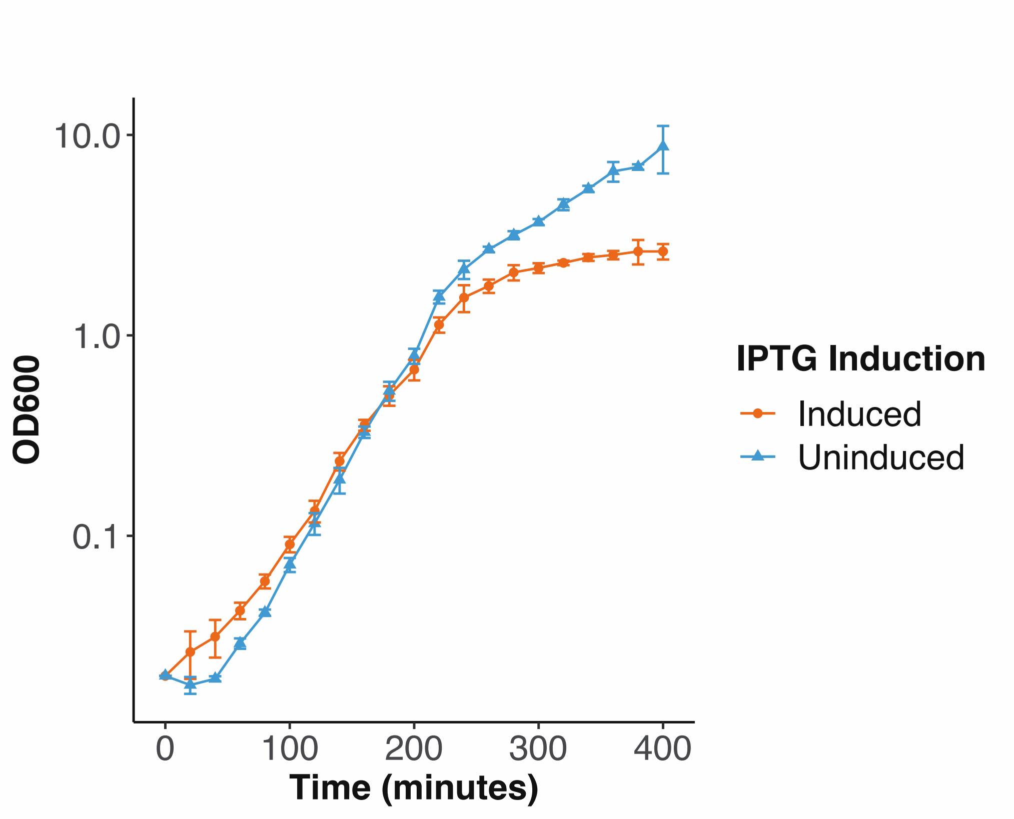

Supplementary Figure S3. IPTG induced expression of *tlyA[K183A*] shows reduced growth compared with uninduced expression. Growth curves show a dominant negative effect when *tlyA*[*K183A*] is induced in a WT background causing a reduction in growth.

References

1. Burby, P. E., Simmons, Z. W., Schroeder, J. W. & Simmons, L. A. Discovery of a dual protease mechanism that promotes DNA damage checkpoint recovery. *PLoS Genet.* **14**, e1007512 (2018).
